# Supplementary material for: Moore’s Law revisited through Intel chip density
Source: PLoS One. 2021 Aug 18;16(8):e0256245. doi: 10.1371/journal.pone.0256245 (PMC8372957; doi:10.1371/journal.pone.0256245)
Supplement: S1 Table — (DOCX) [file pone.0256245.s001.docx]

**S1 Table. Data collected for the analysis is presented here.**

| Year | Transistors (number per CPU) | Density (transistors per mm^2^) |
| --- | --- | --- |
| 1959 | 1 | 0.62 |
| 1961 | 4 | 0.51 |
| 1962 | 9 | 1.0 |
| 1963 | 17 | 1.7 |
| 1964 | 19 | 2.6 |
| 1965 | 14 | 4.0 |
| 1966 | 68 | 13.0 |
| 1967 | 64 | 11.3 |
| 1968 | 192 | 14.2 |
| 1971 | 2300 | 95.8 |
| 1972 | 3500 | 175 |
| 1974 | 6000 | 300 |
| 1976 | 6500 | 325 |
| 1978 | 29000 | 879 |
| 1979 | 29000 | 879 |
| 1982 | 134000 | 2735 |
| 1985 | 275000 | 2644 |
| 1988 | 275000 | 2644 |
| 1989 | 1200000 | 7500 |
| 1990 | 855000 | 8221 |
| 1991 | 1200000 | 19048 |
| 1992 | 1400000 | 22222 |
| 1993 | 3100000 | 10544 |
| 1994 | 3300000 | 20245 |
| 1995 | 5500000 | 36667 |
| 1996 | 3300000 | 36667 |
| 1997 | 7500000 | 57252 |
| 1998 | 27400000 | 152222 |
| 1999 | 44000000 | 550000 |
| 2000 | 44000000 | 550000 |
| 2001 | 44000000 | 550000 |
| 2002 | 55000000 | 550000 |
| 2003 | 169000000 | 927711 |
| 2004 | 169000000 | 1609195 |
| 2005 | 230000000 | 1609195 |
| 2006 | 582000000 | 2320988 |
| 2007 | 820000000 | 3831776 |
| 2008 | 820000000 | 5000000 |
| 2009 | 820000000 | 5000000 |
| 2010 | 1770000000 | 7405858 |
| 2011 | 2270000000 | 7405858 |
| 2012 | 2270000000 | 8750000 |
| 2013 | 1400000000 | 8750000 |
