# Supplementary material for: Moore’s Law revisited through Intel chip density
Source: PLoS One. 2021 Aug 18;16(8):e0256245. doi: 10.1371/journal.pone.0256245 (PMC8372957; doi:10.1371/journal.pone.0256245)
Supplement: Attachment — Submitted filename: Response to Reviewers.docx [file pone.0256245.s002.docx]

Reviewer #1 identifies two drawbacks

1. *The recent analysed data do not include technology nodes from other semiconductor companies except Intel*. The reviewer suggests inclusion of *the data of another semiconductor companies making digital semiconductor transistors from Samsung, TSMC, Global Foundries, IBM, Texas Instrument, SK Hynix, Micron Technology etc .*

The reviewer adds*: If the manufacturing of the semiconductor transistors turn to be to broad for the scope of the paper, the work should focus on the segment of the manufacturing of their choice and clearly state the focus* .

We appreciate the suggestion but the suggestion would turn this paper, already quite long, into a monograph or book. We narrow the focus of the paper in the title, abstract and in the text by stating the inclusion of data from Intel/Fairchild and explaining our choice and challenges of a study of larger scope.

2. . …*the most valuable relation between trends observed in the data and technological changes introduced into technology nodes in order to continue scaling or to continue advancement of the transistor technology is not present* . *…a palette of so-called technology boosters was progressively introduced into the semiconductor manufacturing ranging from the change of Ge to Si, the change of metal interconnects, the change of various dielectrics, device architectures, the amendments of material properties like strain, etc .*

We agree with Reviewer #1 that this history is most interesting. We refer to several historical relationships in the Discussion. We are enthusiasts for such history and have added references to two excellent books, and an IEEE article as well as referencing online materials, which discuss exactly the kinds of developments to which Reviewer #1 refers. The purpose of our analysis is to understand the shape and mathematics of chip evolution; we do not claim to have done original research on particular causes.

3. *Citation to LogletLab software*

We thank the reviewer for bringing this oversight to our attention. The modeling software is located at **logletlab.com**. This has been remedied.

4. *References for 2 statements*

The software citations were moved to the References section and cited in the text. We believe that common statistical procedures need no specific citations, and the original studies are, in any case, referred to in the software documentation.

5. *Clarifications*:

We clarified our decision to not include the R-square statistic and explained the use of AICc for model selection (as opposed to F-tests or comparison of goodness-of-fit measures which do not penalize the model for increased complexity and underestimating overfitting).

6. *Clarify per Automatic Minimum Detection*

This is not core to our results and was omitted.

7. *Delete the word Therefore, start section “We defined…” ; check for any other spacing issues*

We thank the reviewer and it has been corrected. We also made every effort to remove extraneous spacing.
